# Psychometric properties of the Chinese Responsibility Scale in university students in Hong Kong, China

**DOI:** 10.3389/fpsyg.2026.1843854

**Published:** 2026-06-25

**Authors:** Daniel Tan Lei Shek, Chaoran Sun, Janet Tsin Yee Leung, Lindan Tan, Xinyu Guo

**Affiliations:** Department of Applied Social Sciences, The Hong Kong Polytechnic University, Kowloon, Hong Kong SAR, China

**Keywords:** Chinese assessment, psychometric properties, responsibility, university student, value, virtue

## Abstract

**Background:**

This paper reports the development and evaluation of psychometric properties of the Chinese Responsibility Scale (CRS), which was designed to capture the multidimensional nature of responsibility in Chinese young people based on an integration of both Western and Chinese philosophies and conceptual models. Based on an integration of philosophical and scientific literature, we proposed a conceptual framework assessing responsibility across three dimensions: responsibility to oneself, family, and society.

**Methods:**

Study 1 was a content validation study conducted with an expert panel (*N* = 10) based on an initial item pool of 26 items. In Study 2, the 20-item CRS was administered to university students in Hong Kong, China, and assessed across two waves, resulting in data from a large sample (*N* = 963 for the matched sample). Different psychometric properties, including factorial validity, measurement invariance, convergent validity, discriminant validity, and reliability of the total scale and subscales were assessed.

**Results:**

The findings support different forms of validity, measurement invariance, and reliability of the CRS, demonstrating its suitability as an objective tool for evaluating self-reported responsibility among emerging adults in the Chinese cultural context. The theoretical and practical implications of the findings are discussed.

## 1 Introduction

### 1.1 Concept of “Responsibility” in the Western literature

The Western literature defines personal responsibility as a higher-order construct involving the internal endorsement of norms and the intentional coordination of cognition, emotion, and behavior (Jønsson and Fasano, 2025). It reflects the ability to regulate one's own thoughts, feelings, and behavior, along with the willingness to hold oneself accountable for one's choices and consequences (Mergler and Patton, 2007; Mergler, 2017; Jønsson and Fasano, 2025; Holdorf and Greenwald, 2018; Auhagen and Bierhoff, 2002; Lauermann, 2014; Nowell and Boyd, 2014). Corresponding to the concept of “rights,” responsibility is one of the cornerstones of civilization, as it reflects the belief that personal accountability brings societal harmony (Darling-Smith, 2007).

According to the Kantian view (Hill Jr, 1994; Blöser, 2015; Arpaly, 2014), an action can only be considered moral if it is motivated by a sense of duty, and moral practices can also influence the criteria for accountability. While Kantian philosophy defines the “Good Will” as an unconditional moral requirement, individuals often differentiate between strict “duties” (legal or social requirements) and “good deeds” (virtuous actions that are praised but not strictly demanded) in contemporary Western contexts (Arpaly, 2014). This distinction creates a boundary where “responsibility” is often equated with compliance to clear, *a priori* defined duties, whereas “virtue” or “good will” is sometimes perceived as a separate, more voluntary category. Responsibility was later extended to the social dimension (Wray-Lake and Syvertsen, 2011), where social responsibility is regarded as a value orientation underlying one's moral, altruistic, civic, and other behaviors that extend beyond the self.

Recent scholarship suggests that responsibility is best understood as a multidimensional and context-sensitive construct rather than a single, unitary trait. Auhagen and Bierhoff (2002) treated responsibility as a broad social phenomenon embedded in moral, interpersonal, and civic life, while Holdorf and Greenwald (2018) advanced this work by proposing a unified taxonomy in which responsibility manifests through accountability, commitment, concern for others, dependability, initiative, and related forms of moral agency. In educational psychology, Lauermann (2014)'s work further highlights responsibility as a role-specific sense of obligation, especially in relation to important outcomes for others, whereas Nowell and Boyd (2014) conceptualized community responsibility as a psychologically meaningful orientation that motivates individuals to take action on behalf of collective wellbeing. Building on these perspectives, Jønsson and Fasano (2025) defined subjective responsibility as “the personal experience of feeling obligated to take initiative or to take precautions to ensure success and avoid errors concerning a specific valuable target (p. 05),” emphasizing both its internal, felt quality and its target-specific, action-oriented nature. Taken together, these accounts suggest that responsibility involves not only compliance with external duties, but also an internalized sense of obligation that is directed toward valued people, tasks, and social outcomes.

Generally speaking, Western responsibility scales emphasize individual agency and self-control, complemented by a broad but still individually framed notion of social and civic responsibility (Arslan and Wong, 2022; Mergler and Shield, 2016). However, existing Western perspectives on responsibility, which are grounded in individualistic principles, may overlook cultural variations and thus may not be generalizable to collectivistic, non-Western contexts. Indeed, the understanding of responsibility is shaped by cultural contexts, with significant differences between Western and Chinese conceptualizations and interpretations (Ren et al., 2023). Moreover, given rapid societal changes (such as urbanization) in contemporary Chinese society (Kuang et al., 2023), the purpose of this paper is to report the development of a Chinese assessment tool for responsibility through an integration of Chinese and Western cultural perspectives.

### 1.2 The concept of responsibility in the Chinese culture

The Confucian tradition offers a fundamentally different orientation toward responsibility compared to Western perspectives (Ames, 2007). In Chinese ethical traditions–particularly Confucianism–responsibility is not merely an obligation triggered by an external rule. Responsibility in Chinese tends to be blended with a good will rather than be separated. Instead, it is embedded in one's social and relational identity. The concept of Ren (benevolence/human-heartedness) and Yi (righteousness/appropriateness) acts as a guiding compass that renders “responsibility” inseparable from the cultivation of one's character (Ren et al., 2023).

Rather than bounded individual accountability, Confucian responsibility operates as a concentric, expanding moral obligation. For example, The Great Learning (大学; da xue) articulates the progression of self-cultivation, family management, state governance, and bringing peace to all under heaven (修身,齐家,治国,平天下; xiu shen, qi jia, zhi guo, ping tian xia) (Ding et al., 2024). Responsibility begins not from an isolated individual but from the harmony of human beings with nature and from blood-based kinship, giving rise to principles of ritual and fitness (礼仪; li yi), with filial piety (孝道; xiao dao) at the core (Ren, 2008). Moreover, traditional Chinese cultural values emphasize faithfulness and loyalty to others, proactive altruistic responsibility, and a community-oriented, interdependent self (Ren et al., 2023). Based on a systematic comparison of Confucian and Western moral responsibility, Jing and Doorn (2020) noted that Confucianism treats responsibility more like an obligation, in contrast to Kantian or utilitarian frameworks that rely on the principle of autonomous moral agency.

Responsible actions in contemporary Chinese society manifest as a mixture of traditional Chinese cultural and Western individualistic values. One of the major targets of responsibility is one's original family. Rooted in filial piety obligations, responsibility is linked with taking care of or even sacrificing for one's family (Qi, 2015). Chinese thought treats the family as the basic unit of society, contrary to Kantian and utilitarian perspectives, which prioritize independence. Being a good child or parent is understood as the starting point for being a good citizen and building social order, as stated in Yijing (正家而天下定矣; zheng jia er tian xia ding yi) . Chinese values also emphasize responsibility to society, particularly through effective governance. In Confucian thought, the capacities for governing oneself, managing the family, and governing the state are seen as continuous and mutually interdependent (Jing and Doorn, 2020; Jiang, 2002; Ding et al., 2024), which contrasts with Kantian accounts that focus on the moral agency of individuals abstracted from familial-political role continuity (Hill Jr, 1994; Blöser, 2015). Therefore, responsibility to one's family was adopted as one important responsibility dimension in the current study, which is not typically highlighted in the Western conceptions.

### 1.3 Research gaps in the literature leading to the current study

Responsibility has been recognized as related to better academic and psychosocial outcomes (e.g., academic achievement, psychological health, and future career planning). Responsibility for learning and agentic engagement mediate the effects of supportive school environments on academic achievement and career decision-making self-efficacy (Mameli et al., 2019), suggesting that when students feel obligated and accountable for their own learning, they perform better and feel more capable of planning their futures. Social responsibility values are associated with a greater sense of community, more prosocial behavior, which, in turn, higher wellbeing during adolescence (Bartolo et al., 2023). Longitudinal data on Chinese youth show that a stronger sense of responsibility to parents, embedded in family obligation norms, predicts greater delay of gratification in the learning process, more constructive responses to academic failure, less secure attachment failure, and more secure attachment to parents over time (Yang et al., 2025). Recent Chinese scales of personal and social responsibility–developed for college students, university students, community residents, and school settings–demonstrate that responsibility is positively related to trust, prosocial behavior, and community identity (Wong and Liang, 2020; Ren et al., 2023; Liu et al., 2020).

Although prior studies have consistently highlighted the developmental significance of responsibility, several conceptual gaps remain in the existing literature regarding the conceptualization and assessment of responsibility among young people. First, Western measures cover personal and social responsibilities, but pay less attention to family responsibility. Second, some Chinese responsibility measures also cover self- and social responsibility, but place less emphasis on family responsibility. For example, the Chinese College Student Personal Responsibility Scale (CCSPRS) was created to capture the personal responsibility of Chinese college students (Ren et al., 2023). For social responsibility, the Chinese University Students' Social Responsibility Scale (CUSSRS) defines social responsibility as a psychological trait of consciously assuming one's role as a citizen and includes four dimensions: nation responsibility, nature responsibility, others responsibility, and organization responsibility, all embedded in students' everyday roles (Liu et al., 2020). Wong and Liang (2020) developed the Personal and Social Responsibility Scale tailored for Chinese high school students that assesses eight aspects, such as respecting others, participation and effort, self-direction, caring, and value, again reflecting a strong focus on relational duties and collective wellbeing. However, previous Chinese responsibility scales did not integrate responsibility toward the family into their measures. Third, there are no studies examining the psychometric properties of responsibility assessment tools across time.

Methodologically, there are also several gaps in the Chinese scientific literature. First, no systematic attempt has been made to develop and validate Chinese measures of responsibility. We conducted a targeted search of the APA PsycINFO database using the Boolean query: title(Chinese) AND title(Responsibility) AND (title(Scale) OR title(Assess*)). This search yielded only 16 results, none of which provided a comprehensive assessment of responsibility. The few relevant scales identified included Liu et al. (2020)'s CUSSRS and Ren et al. (2023)'s CCSPRS. Second, there is a lack of longitudinally validated scales on Chinese responsibility for emerging adults and university students. Although the CCSPRS (Ren et al., 2023) and CUSSRS (Liu et al., 2020) showed, good reliability and model fit, the related evidence was based on cross-sectional findings only. Third, the sample sizes of the existing studies on emerging adult responsibility were relatively small. For example, Ren et al. (2023) used a sample size of 301 and Liu et al. (2020) used a sample size of 646.

Against the context above, we developed and longitudinally validated the Chinese Responsibility Scale (CRS) to assess responsibility in Chinese emerging adults from the perspectives of self, family, and society following the scale development procedure recommended by Hoyle et al. (2025). In Study 1, an initial item pool was generated through a review of both Western and Chinese literature, and content validity was established through expert panel evaluation using the Content Validity Index (CVI). Following recommended procedures for scale construction (Boateng et al., 2018), our aim was first to ensure that the items were clear, relevant, and conceptually appropriate, and then rigorously test the reliability and validity of the instrument before interpreting its scores. Content validity was addressed through theory-based item generation and expert review to check that the scale covered the target construct without omitting key aspects or introducing extraneous content, with revisions guided by the CVI and expert feedback (Zamanzadeh et al., 2015).

In Study 2, the psychometric properties of the CRS were examined in details using a longitudinal two-wave design following Hoyle et al. (2025)'s scale validation procedure. According to Hoyle et al. (2025), validity and reliability should be assessed to ensure that the scale is valid. Specifically, we investigated whether the proposed three-factor structure demonstrated adequate fit and factorial validity (Research Question 1), whether the factor structure and scores remained stable over time through measurement invariance testing (Research Question 2), and whether the scale and its subscales evidenced convergent validity (Research Question 3) and discriminant validity (Research Question 4). Convergent and discriminant validity were examined jointly as evidence of construct validity, with the former indicating appropriate associations with related constructs and the latter demonstrating separation from theoretically distinct constructs (Hamid et al., 2017). Longitudinal reliability of the scale and subscales across time was also assessed (Research Question 5). Reliability analyses (e.g., internal consistency) were then used to confirm that the items yielded stable, consistent scores, given that non-reproducible measures cannot support trustworthy inferences.

Together, these two studies sought to establish a psychometrically sound and culturally informed measure of responsibility suitable for use among Chinese emerging adults.

## 2 Study 1: item generation and content validation

### 2.1 Methodology

The research team developed the CRS to evaluate the sense of responsibility of the students, drawing on reviews of the scientific and Chinese literature. The initial draft comprised 26 items across three dimensions: self-, family-, and social responsibility, developed by a team of psychologists and social workers with substantial experience in scale development. Responses were captured on a 6-point Likert scale (1 = most dissimilar, 6 = most similar). The Responsibility for Self-Subscale was adapted from the Houghton and Neck (2002) Self-Leadership Questionnaire and the Mergler and Shield (2016) Personal Responsibility Scale. The Responsibility for the Society Subscale was informed by Witvliet et al. (2023)'s Accountability Scale. The Responsibility for Family Subscale was theoretically informed by the findings of Leung et al. (2023) regarding the significant mediating role of filial piety among Chinese adolescents. A 10-member expert panel evaluated the clarity and relevance of the items.

### 2.2 Results and discussion

Based on the responses to the content validation questionnaire, the research team calculated the CVI. Following the recommendations of Zamanzadeh et al. (2015), the results in Table 1 indicate that the CVI values for the items are generally very high. The experts also provided feedback on the wording and appropriateness of the items. Feedback such as “Pleasing others did not seem relevant to responsibility” and “How does an item mentioning siblings apply to a single-child family” were incorporated to revise the draft items and options. Based on the CVI and expert feedback, we modified or removed some items following the best practices for scale development (Boateng et al., 2018). Consequently, the final version comprises 20 items (see Table 2). The traditional Chinese item content used for content validation and data collection is in Supplementary Table S1.

**Table 1:** Content validity index.

Content validity indices	(1) Relevance		(2) Clarity	
Range	0.60–1.00	0.50–1.00	0.80–1.00	0.79–1.00
% of items meets standard (i.e. I-CVI = 0.78; Kappa = 0.75)	92.31%	92.31%	100%	100%
S-CVI (average)=	0.93		0.96	
S-CVI (proportion)=	0.93		0.96	
Average Kappa =	0.92		0.96	
	**(3) Representativeness**			
**Domains**	**I-CVI**	**Multi-rater modified Kappa**		
1. Responsibility for the self	1.00	1.00		
2. Responsibility for the family	1.00	1.00		
3. Responsibility for the society	1.00	1.00		
Range	1.00	1.00		
% of items meets standard (i.e. I-CVI = 0.78; Kappa = 0.75)	100%	100%		
S-CVI (average)=	1.00			
S-CVI (proportion)=	1.00			
Average Kappa =	1.00			

I-CVI, item-level content validity index; S-CVI, scale-level content validity index. 80% of experts (8 out of 10 experts) admitted that 3 domains were enough to represent the concept of responsibility.

**Table 2:** Item content, mean, standard deviation, skewness, and kurtosis of the items across two time points (*N* = 963).

Item content	Wave 1				Wave 2			
	Mean	Std. deviation	Skewness	Kurtosis	Mean	Std. deviation	Skewness	Kurtosis
1. (Se) I understand the importance of improving my self-cultivation	5.15	0.83	−1.15	2.73	5.11	0.81	−0.74	0.89
2. (Fa) I understand my family's expectations of me	4.84	0.98	−1.03	1.69	4.85	0.92	−0.85	1.24
3. (So) I understand the role I play in society	4.51	1.12	−0.78	0.70	4.49	1.10	−0.66	0.36
4. (Fa) I understand the role I play in my family	4.70	0.99	−0.76	1.03	4.71	0.96	−0.67	0.71
5. (So) I agree that everyone should care about society	4.71	1.15	−0.94	0.82	4.72	1.03	−0.76	0.70
6. (Fa) I think it is very important to take on family responsibilities	4.83	1.06	−1.02	1.30	4.80	1.00	−0.95	1.37
7. (Fa) Family harmony is important	5.17	0.99	−1.46	2.61	5.15	0.88	−1.09	1.79
8. (Se) Everyone should follow social norms	4.89	1.07	−1.00	1.08	4.93	0.98	−1.03	1.61
9. (So) I care about whether what I do has a positive impact on society	4.47	1.21	−0.66	0.13	4.50	1.14	−0.67	0.22
10. (Fa) I am willing to share my family members' worries	4.59	1.10	−0.78	0.58	4.65	1.03	−0.81	0.98
11. (So) I am happy to see a harmonious society	5.20	0.85	−1.11	1.98	5.20	0.81	−0.91	1.41
12. (Fa) I often help my family with household chores	4.22	1.29	−0.54	−0.16	4.34	1.21	−0.66	0.27
13. (Se) Everyone should take responsibility for their words and actions	5.20	0.85	−1.25	2.78	5.18	0.81	−0.88	1.16
14. (Fa) I am willing to take care of my family members' emotions	4.68	1.09	−0.81	0.66	4.65	1.04	−0.73	0.55
15. (So) I often care about what happens in society	4.46	1.14	−0.61	0.15	4.47	1.10	−0.53	0.03
16. (Se) I am willing to better myself to fulfill my responsibilities more effectively	4.85	0.95	−0.86	1.40	4.84	0.89	−0.78	1.40
17. (Fa) I am willing to take care of my elderly parents	4.92	1.02	−1.00	1.25	4.89	0.95	−0.89	1.17
18. (So) I am willing to do things for my community to make it better	4.62	1.07	−0.82	1.08	4.64	0.98	−0.56	0.58
19. (Se) I will do my best to carry out my duties	5.11	0.89	−1.19	2.50	5.04	0.83	−0.72	0.69
20. (Fa) I am willing to take on family responsibilities	4.80	1.03	−0.85	1.08	4.78	0.99	−0.92	1.40

Se, Self; Fa, Family; So, Society.

## 3 Study 2: psychometric properties of the CRS in a longitudinal study

### 3.1 Research questions and hypotheses

Using the 20-item Chinese Responsibility Scale (CRS), we formulated several research questions and proposed related hypotheses.

Research Question 1: Are the three proposed dimensions intrinsic to the CRS supported by the longitudinal data?

- Hypothesis 1a: The related items would load significantly on the proposed factors.
- Hypothesis 1b: Model fit indices would be acceptable according to established criteria with the comparative fit index (CFI) ≥0.90, Tucker-Lewis Index (TLI)≥0.90 (Hoyle, 1995), root-mean square error of approximation (RMSEA) ≤ 0.08 (Browne and Cudeck, 1992), and standardized root mean square residual (SRMR) ≤ 0.08 (Ximénez et al., 2022; Kline, 2023).

Research Question 2: Is the CRS underlying factor structure longitudinally stable?

- Hypothesis 2: The three-factor CRS model structure would exhibit invariance over the two waves.

Research Question 3: Do the CRS total scale and subscales demonstrate convergent validity?

- Hypothesis 3a: Correlations between theoretically relevant measures and the total scale would be significant.
- Hypothesis 3b: Correlations between theoretically relevant measures and the subscales would be significant.
- Hypothesis 3c: The Average Variance Extracted (AVE) for each factor would be ≥0.50.

Research Question 4: Do the CRS total scale and subscales demonstrate discriminant validity?

- Hypothesis 4a: Correlations between theoretically irrelevant items and the CRS total scale would be low.
- Hypothesis 4b: Correlations between theoretically irrelevant items and the CRS subscales would be low.
- Hypothesis 4c: The square root of AVE for each construct would exceed the factor correlations with other constructs.
- Hypothesis 4d: Heterotrait-monotrait correlation (HTMT) ratios would be ≤ 0.90 between all subscales.

Research Question 5: Are the CRS and subscales longitudinally reliable?

- Hypothesis 5a: Composite reliability of the subscales would be ≥0.70.
- Hypothesis 5b: Cronbach's α and McDonald's ω of the subscales and total scale would be ≥0.70.
- Hypothesis 5c: Inter-item correlations of the subscales and total scale would be ≥0.30 across the two waves.

### 3.2 Materials and methods

#### 3.2.1 Participants and study design

**Figure 1:**
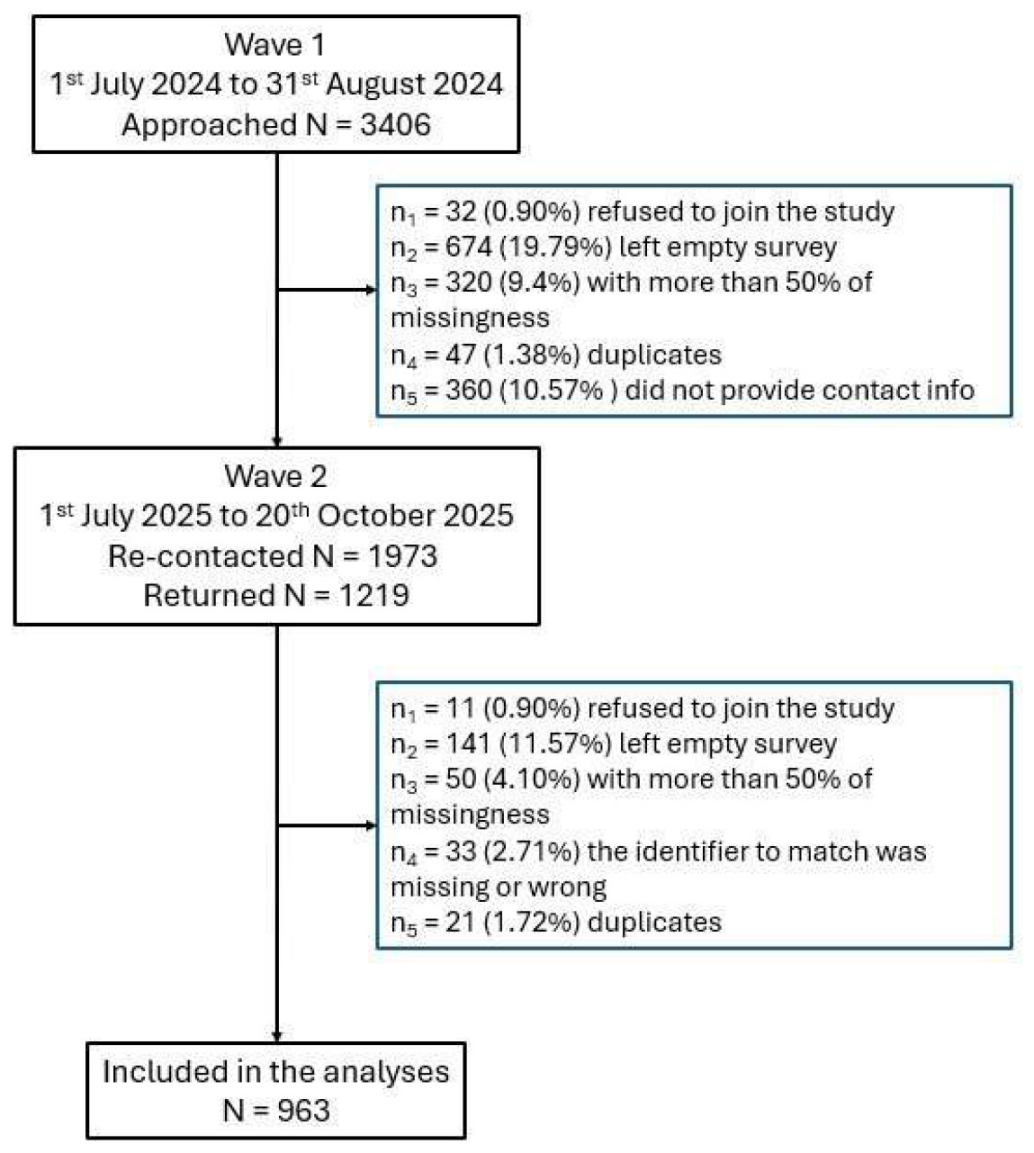
Details on the data collection procedure.

Participants were recruited via convenience sampling from eight public and two private universities in Hong Kong, China, by student helpers. All participants received information about confidentiality and their right to withdraw or decline inclusion at any point. Completed surveys with over 50% of items filled earned participants a HK$100 supermarket voucher. Unique codes facilitated cross-wave matching. Wave 1 data collection spanned July 1 to August 31, 2024 (*N* approached = 3,406; n retained for Wave 2 = 1,973 after excluding refusals, non-responses, excessive missing data, missing contacts, and duplicates). Wave 2 data were gathered from July 1 to October 20, 2025 (*N* returned = 1,219; *n* matched to Wave 1 with >70% completeness = 963). See Figure 1 for full details. Ethics approval was secured from the corresponding author's affiliated institution.

#### 3.2.2 Instruments

Participants provided demographic information including age, gender, parental education and marital status, academic year, low-income/social security status, and Hong Kong permanent residency. Participants indicated their extent of similarity with the item content using a 6-point Likert scale from “1” (not at all similar) to “6” (very similar). The total scores were averaged. Besides the CRS, convergent validity was examined using the Contemporary Filial Piety Scale (CFPS) (Lum et al., 2016), Adolescent Responsible Behavior Scale (ARBS) (Zhou et al., 2021), five items from Scale of Student Egocentrism (SSE) (Shek et al., 2014), 15 items from Student Moral Character Scale (SMCS) (Shek et al., 2021), the three-item Moral Competence Scale (MC) (Shek and Zhu, 2018, 2019), and four items from Positive Relationship with Others Scale (PRO) (Ryff, 1989). All external measures showed acceptable reliability (CFPS α_*T*1_ = 0.89, α_*T*2_ = 0.91; ARBS α_*T*1_ = 0.88, α_*T*2_ = 0.89; SSE α_*T*1_ = 0.78, α_*T*2_ = 0.75; SMCS α_*T*1_ = 0.87, α_*T*2_ = 0.87; MC α_*T*1_ = 0.70, α_*T*2_ = 0.76; PRO α_*T*1_ = 0.77, α_*T*2_ = 0.76).

Additional measures of values including Respect, Care, Acceptance, and Integrity–previously content-validated, were included for convergent validity and demonstrated strong reliability (Care α_*T*1_ = 0.94, α_*T*2_ = 0.95; Acceptance α_*T*1_ = 0.94, α_*T*2_ = 0.94; Respect α_*T*1_ = 0.94, α_*T*2_ = 0.93; Integrity α_*T*1_ = 0.92, α_*T*2_ = 0.93). Participants indicated their extent of similarity with the item content using a 6-point Likert scale, ranging from “1” (not at all similar) to “6” (very similar). Four prosocial attitudes items (“I care about people in society who have encountered misfortune”; “If given the opportunity, I would participate in volunteer work”; “I agree that everyone should be bound by the law”; “If I do something wrong, I would feel ashamed”) plus a single item (“How do you rate your morality?”) were also assessed for convergent validity. Four theoretically unrelated items were used to assess discriminant validity: (a) liking Korean movies, (b) liking basketball, (c) agreement that Hong Kong has a comfortable climate, and (d) liking ice cream. Participants rated these items using a 4-point Likert scale from “1” (strongly dislike/disagree) to “4” (strongly like/agree).

#### 3.2.3 Data analysis

Descriptive and Pearson correlation analyses were conducted using SPSS Version 30 (IBM Corp., 2025). Structural equation modeling (SEM) and confirmatory factor analysis (CFA) assumptions were met, including normality assessed by the absolute values of skewness (< 2) and kurtosis (< 7) thresholds for observed variables (Curran et al., 1996; Kline, 2023). Modeling and invariance testing were performed in Mplus Version 8.3 (Muthén and Muthén, 2019), with missing data handled via full-information maximum likelihood (FIML).

Construct validity, indexed by factorial validity (Hypothesis 1a), was tested through CFA, treating observed items as indicators of correlated latent factors, with waves modeled as a grouping variable (Keller et al., 1998). The two-wave data were analyzed in a single model yielding unified fit indices (Hypothesis 1b). The model comparisons utilized AIC and BIC, where Δ*AIC*>10 or Δ*BIC*>10 favored the lower value (Burnham and Anderson, 2004). Initial fit evaluation used the chi-squared test (non-significant p = excellent fit), although its large-sample sensitivity prompted reliance on absolute indices (SRMR, RMSEA) and incremental indices, including CFI and TLI (Kline, 2023). Acceptable fit required TLI/CFI >0.90 (Hoyle, 1995), SRMR < 0.08 (Kline, 2023; Ximénez et al., 2022), and RMSEA < 0.08 (Browne and Cudeck, 1992).

Measurement invariance (Hypothesis 2) across waves was evaluated via configural, metric, and scalar models in Mplus Version 8.3 (Muthén and Muthén, 2019). Invariance was determined by |Δ*CFI*| (< 0.002), |Δ*SRMR*| (< 0.030), and |Δ*RMSEA*|(< 0.010) between nested models (Meade et al., 2008; Kline, 2023). We also explored invariance across gender and local vs. non-local status.

Convergent validity was confirmed by (1) significant Pearson correlations between CRS total scale and subscale scores and theoretically related constructs (Hypotheses 3a and 3b) and (2) AVE>0.50 for factors and total scale and subscales across waves (Hypothesis 3c; Hamid et al., 2017). Discriminant validity of the total scale used correlations between CRS totals and unrelated filler items requiring (1) low correlations with unrelated items (Hypotheses 4a and 4b), (2) square root AVE exceeding inter-factor correlations (Hypothesis 4c), and (3) HTMT ratio < 0.90 (Hypothesis 4d; Hamid et al., 2017).

Reliability included composite reliability ≥ 0.70 (Hypothesis 5a; Raykov, 2004). The internal consistency for the subscales and full CRS was assessed using Cronbach's α, McDonald's ω(≥0.70 acceptable; Hypothesis 5b), and mean inter-item correlations with 0.30–0.70 as optimal (Davenport et al., 2015; Dunn et al., 2014; Kruger et al., 2023; Liu et al., 2023).

#### 3.2.4 Attrition analysis

Participants were less likely to participate in Wave II if they were male (χ^2^(1) = 8.04, *p* < 0.01), younger (χ^2^(2) = 6.50, *p* < 0.05), and born in Hong Kong (χ^2^(3) = 8.79, *p* < 0.05). There was no significant distributional difference in whether their household received low-income social welfare (χ^2^(2) = 0.59, *p* = 0.74), parental marriage status (χ^2^(4) = 1.45, *p* = 0.84), father's (χ^2^(7) = 6.35, *p* = 0.50) and mother's (χ^2^(7) = 5.76, *p* = 0.57) highest education background. No significant differences were found in the CRS total score (*t*(1970) = −0.77, *p* = 0.44).

### 3.3 Results

Descriptive analysis results are shown in Table 3. The majority of the participants were female (72.1%) and aged 19–24 years (89.4%). Most were identified as local students (62.4%). Regarding parental background, nearly half reported at least one parent with an Associate Degree or higher (45.6%), while 81.9% indicated their parents were married. Only a small proportion were from low-income families (2.4%). Participants were relatively evenly distributed across year groups, with 34.9% sophomores, 30.0% juniors, and 34.5% seniors. As the skewness and kurtosis of the items met the threshold of normality (Table 2), the modeling analysis proceeded using a maximum likelihood robust (MLR) estimator.

**Table 3:** Demographic data (*N* = 963).

Demographics	*n* (%)
Gender	
Female	694 (72.1%)
Male	265 (27.5%)
Age	
18	21 (2.2%)
19–24	861 (89.4%)
25 or above	71 (7.4%)
Identity status	
Local	601 (62.4%)
Non-local	356 (36.9%)
Parent's highest education background	
Associate degree, bachelor's, or above	439 (45.6%)
High school	155 (16.1%)
Middle school	254 (26.4%)
Primary school or less	74 (7.7%)
Not sure	40 (4.2%)
Parent's marital status	
Married	789 (81.9%)
Not married	168 (17.4%)
Being categorized as low-income family	
No	907 (94.2%)
Yes	23 (2.4%)
Not sure	31 (3.2%)
**Year group**	
Sophomore	336 (34.9%)
Junior	289 (30.0%)
Senior	332 (34.5%)

#### 3.3.1 Factorial validity

**Figure 2:**
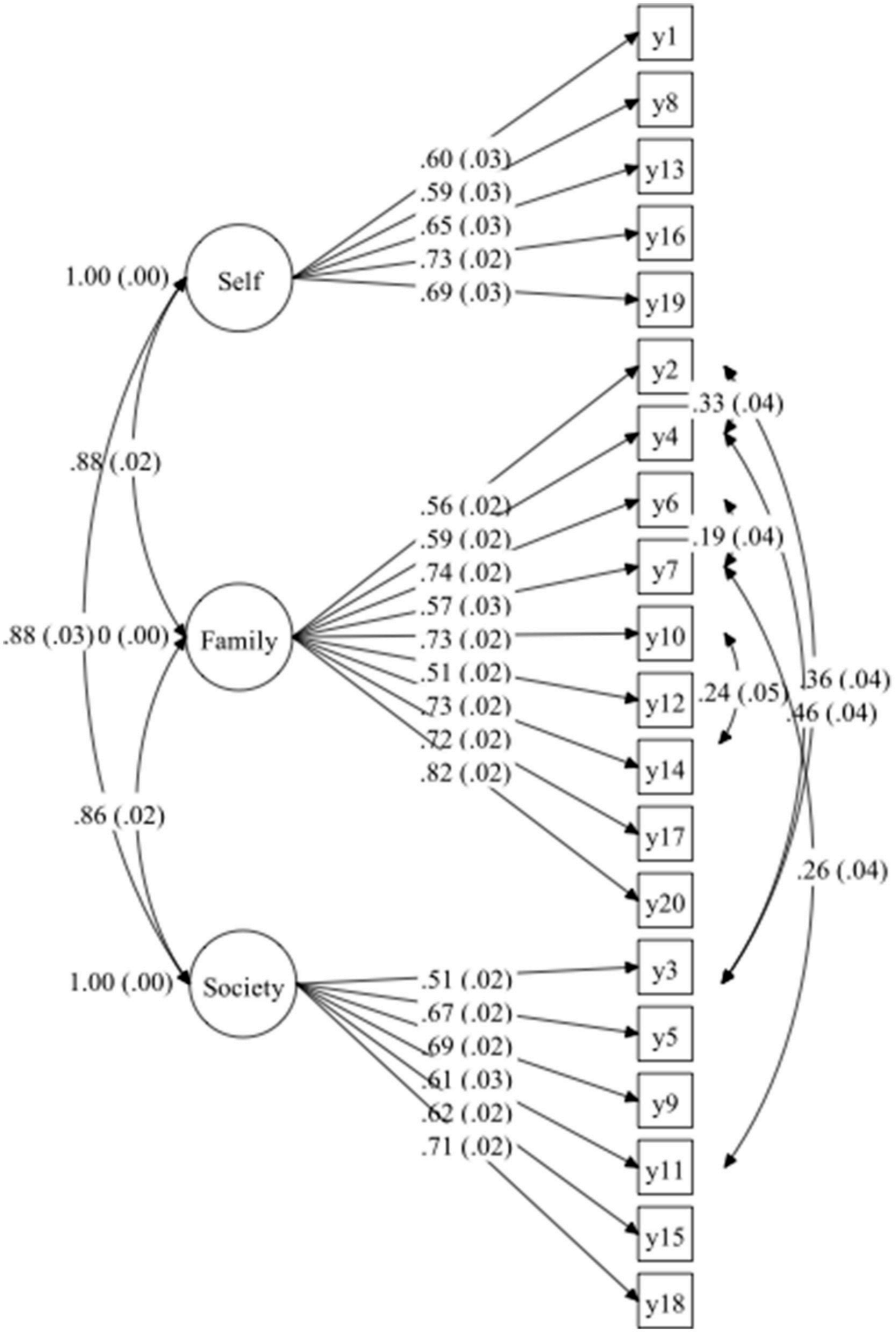
The model diagram of CFA with standardized pathway estimates (Time 1).

The EFA results suggested a four-factor model which was empirically supported by CFA (*CFI* = 0.91, *TLI* = 0.89, *RMSEA* = 0.07, *SRMR* = 0.04). However, the indicators suggested by the EFA did not have sufficient theoretical support. In contrast, the three-factor CFA model with items specified consistent with the content validation, achieved the best model fit indices (χ^2^(356) = 1425.97, *p* < 0.001;*CFI* = 0.91, *TLI* = 0.90, *RMSEA* = 0.06, *SRMR* = 0.05) compared to models based on the EFA results and a one-factor model (Table 4). Six pairs of item residuals were correlated based on similar wording, modification indices, and agreement between the analysts. Items 2, 3, and 4 (all containing “understand”), Items 6 and 7 (“important”), Items 7 and 11 (“harmony”), and Items 10 and 14 (“willing”) showed correlated residuals which can be explained by shared item wording or method effects. The standardized factor loadings ranged from 0.51 to 0.82, all significant at *p* < 0.001. Given the solid theoretical foundation and adequate model fit, the three-factor model aligned with content validation was retained for further analysis. Therefore, Hypotheses 1a and 1b were supported. The detailed model diagram based on Wave 1 data is presented in Figure 2.

**Table 4:** CFA model comparisons with time as grouping (*N* = 963).

CFA model fit	AIC	BIC	CFI	TLI	RMSEA	SRMR
Model 1: Three-factor (based on content validation)	47,396.17	47,732.21	0.91	0.90	0.06	0.05
Model 2: Three-factor (based on EFA)	47,561.46	47,868.27	0.89	0.87	0.08	0.05
Model 3: Four-factor (based on EFA)	47,391.46	47,712.89	0.91	0.89	0.07	0.04
Model 4: One-factor	48,101.50	48,393.71	0.83	0.81	0.06	0.10

#### 3.3.2 Measurement invariance

Measurement invariance testing supported configural, weak, and strong invariance between the time points, as indicated by minimal changes in CFI ( ≤ 0.002) and RMSEA ( ≤ 0.001). However, strict invariance was not supported, given a significant chi-square difference (*p* < 0.001) and a notable decline in model fit (Δ*CFI* = 0.004, Δ*RMSEA* < 0.001). Overall, the results suggest that factor pattern, factor loadings, and intercepts were equivalent across groups, but residual variances differed. Thus, Hypothesis 2 was accepted in terms of configural, weak and strong invariance. However, the scale only demonstrated configural and weak measurement invariance across gender and local/non-local status. Detailed results are presented in Table 5.

**Table 5:** Measurement invariance (MI) of the CRS across two time points, gender, and local/non-local status (*N* = 963).

Invariance model	Global fit indices							Models	Δ **test**			
	χ^2^	df	*p*	TLI	CFI	RMSEA	SRMR		Δ χ^2^	*p*	ΔCFI	ΔRMSEA
Time invariance												
1. Configural model	1,368.45	322	< 0.001	0.890	0.907	0.058	0.051	–	–	–	–	–
2. Weak MI	1,387.74	339	< 0.001	0.896	0.907	0.057	0.052	2 vs. 1	9.74	0.91	0.000	0.001
3. Strong MI	1,425.97	356	< 0.001	0.899	0.905	0.056	0.053	3 vs. 2	20.3	0.261	0.002	0.001
4. Strict MI	1,488.77	376	< 0.001	0.900	0.901	0.055	0.065	4 vs. 3	65.9	< 0.001	0.004	< 0.001
Gender invariance												
1. Configural model	1,371.50	322	< 0.001	0.888	0.905	0.058	0.050	–	–	–	–	–
2. Weak MI	1,398.06	339	< 0.001	0.892	0.904	0.057	0.056	2 vs. 1	21.67	0.20	0.001	0.001
3. Strong MI	1,490.02	356	< 0.001	0.890	0.897	0.058	0.060	3 vs. 2	104.73	< 0.001	0.007	0.001
Local status invariance												
1. Configural model	1,406.18	322	< 0.001	0.832	0.847	0.057	0.058	–	–	–	–	–
2. Weak MI	1,434.96	339	< 0.001	0.836	0.847	0.057	0.063	2 vs. 1	22.13	0.18	< 0.001	< 0.001
3. Strong MI	1,499.73	356	< 0.001	0.837	0.844	0.057	0.064	3 vs. 2	61.07	< 0.001	0.003	< 0.001

#### 3.3.3 Convergent validity

Regarding the convergent validity of the CRS (Tables 6, 7), three sources of evidence were considered. First, total scores were significantly correlated with the CFPS (r ranged from 0.44 to 0.60, *p* < 0.01), ARBS (r ranged from 0.44 to 0.65, *p* < 0.01), SMCS (*r* ranged from 0.43 to 0.67, *p* < 0.01), MC (*r* ranged from 0.37 to 0.48, *p* < 0.01), and PRO (*r* ranged from 0.39 to 0.52, *p* < 0.01). Only the correlations between total scores at Wave 2 and the SSE were insignificant (*r* ranged from −0.06 to −0.02, *p*>0.05). Second, total scores were significantly correlated with the other value scales, including Respect (*r* ranged from 0.38 to 0.67, *p* < 0.01), Care (*r* ranged from 0.41 to 0.70, *p* < 0.01), Acceptance (*r* ranged from 0.34 to 0.63, *p* < 0.01), and Integrity (*r* ranged from 0.44 to 0.74, *p* < 0.01). Third, total scores were significantly correlated with the items used to test convergent validity (*r* ranged from 0.21 to 0.41, *p* < 0.01). Therefore, Hypothesis 3a was accepted.

**Table 6:** Correlations between the CRS whole and subscales with CFPS, ARBS, and other measures of values for convergent validity (*N* = 963).

Factors	CFPS	CFPS	ARBS	ARBS	Respect	Respect	Care	Care	Accept	Accept	Integrity	Integrity
	W1	W2	W1	W2	W1	W2	W1	W2	W1	W2	W1	W2
Self W1	0.44^**^	0.32^**^	0.52^**^	0.34^**^	0.67^**^	0.38^**^	0.62^**^	0.32^**^	0.59^**^	0.29^**^	0.71^**^	0.41^**^
Family W1	0.62^**^	0.48^**^	0.60^**^	0.41^**^	0.59^**^	0.37^**^	0.61^**^	0.38^**^	0.53^**^	0.28^**^	0.66^**^	0.42^**^
Society W1	0.44^**^	0.35^**^	0.63^**^	0.44^**^	0.60^**^	0.38^**^	0.67^**^	0.41^**^	0.63^**^	0.36^**^	0.68^**^	0.45^**^
Self W2	0.36^**^	0.46^**^	0.39^**^	0.51^**^	0.36^**^	0.64^**^	0.38^**^	0.62^**^	0.32^**^	0.59^**^	0.39^**^	0.68^**^
Family W2	0.52^**^	0.65^**^	0.45^**^	0.60^**^	0.33^**^	0.54^**^	0.39^**^	0.63^**^	0.31^**^	0.53^**^	0.39^**^	0.62^**^
Society W2	0.39^**^	0.47^**^	0.49^**^	0.63^**^	0.35^**^	0.57^**^	0.44^**^	0.66^**^	0.38^**^	0.62^**^	0.41^**^	0.67^**^
Total W1	0.57^**^	0.44^**^	0.65^**^	0.44^**^	0.67^**^	0.41^**^	0.70^**^	0.41^**^	0.63^**^	0.34^**^	0.74^**^	0.47^**^
Total W2	0.49^**^	0.60^**^	0.49^**^	0.65^**^	0.38^**^	0.63^**^	0.44^**^	0.70^**^	0.37^**^	0.63^**^	0.44^**^	0.71^**^

CFPS, Contemporary Filial Piety Scale; ARBS, Adolescent Responsible Behavior Scale; ^**^p < 0.01.

**Table 7:** Correlations between the CRS whole and subscales and convergent scales and items (*N* = 963).

Convergent scales & items	Self	Family	Society	Self	Family	Society	Total	Total
	W1	W1	W1	W2	W2	W2	W1	W2
SSE W1	−0.07^*^	−0.05	−0.08^*^	−0.07^*^	−0.05	−0.04	−0.07^*^	−0.06
SSE W2	−0.10^**^	−0.08^*^	−0.11^**^	−0.05	−0.03	0.01	−0.10^**^	−0.02
SMCS W1	0.60^**^	0.61^**^	0.64^**^	0.36^**^	0.40^**^	0.44^**^	0.67^**^	0.45^**^
SMCS W2	0.35^**^	0.39^**^	0.43^**^	0.56^**^	0.54^**^	0.59^**^	0.43^**^	0.62^**^
MC W1	0.47^**^	0.42^**^	0.41^**^	0.40^**^	0.34^**^	0.38^**^	0.47^**^	0.40^**^
MC W2	0.37^**^	0.31^**^	0.34^**^	0.53^**^	0.40^**^	0.44^**^	0.37^**^	0.48^**^
PRO W1	0.43^**^	0.51^**^	0.52^**^	0.37^**^	0.44^**^	0.43^**^	0.54^**^	0.46^**^
PRO W2	0.29^**^	0.38^**^	0.37^**^	0.40^**^	0.50^**^	0.49^**^	0.39^**^	0.52^**^
How do you rate your morality (W1)?	0.33^**^	0.31^**^	0.35^**^	0.30^**^	0.27^**^	0.31^**^	0.36^**^	0.32^**^
How do you rate your morality (W2)?	0.30^**^	0.30^**^	0.33^**^	0.32^**^	0.32^**^	0.36^**^	0.34^**^	0.37^**^
I care about people in society who have encountered misfortune (W1).	0.31^**^	0.33^**^	0.43^**^	0.28^**^	0.29^**^	0.40^**^	0.39^**^	0.36^**^
If given the opportunity, I would participate in volunteer work (W1).	0.32^**^	0.35^**^	0.44^**^	0.26^**^	0.28^**^	0.38^**^	0.41^**^	0.34^**^
I agree that everyone should be bound by the law (W1)	0.40^**^	0.29^**^	0.26^**^	0.32^**^	0.22^**^	0.21^**^	0.34^**^	0.27^**^
If I do something wrong, I would feel ashamed (W1)	0.42^**^	0.34^**^	0.29^**^	0.30^**^	0.24^**^	0.22^**^	0.38^**^	0.27^**^
I care about people in society who have encountered misfortune (W2)	0.20^**^	0.21^**^	0.32^**^	0.36^**^	0.33^**^	0.43^**^	0.27^**^	0.40^**^
If given the opportunity, I would participate in volunteer work (W2)	0.18^**^	0.24^**^	0.32^**^	0.30^**^	0.35^**^	0.43^**^	0.28^**^	0.40^**^
I agree that everyone should be bound by the law (W2)	0.23^**^	0.19^**^	0.18^**^	0.43^**^	0.30^**^	0.29^**^	0.21^**^	0.36^**^
If I do something wrong, I would feel ashamed (W2)	0.30^**^	0.27^**^	0.28^**^	0.45^**^	0.34^**^	0.32^**^	0.31^**^	0.39^**^

SSE, Scale of Student Egocentrism; SMCS, Student Moral Character Scale; MC, Moral Competence Scale; PRO, Positive Relationship with Others. ^**^p < 0.01; ^*^p < 0.05.

Regarding the subscales, scores were significantly correlated with the CFPS (*r* ranged from 0.32 to 0.65, *p* < 0.01), ARBS (*r* ranged from 0.34 to 0.63, *p* < 0.01), SMCS (*r* ranged from 0.35 to 0.64, *p* < 0.01), MC (*r* ranged from 0.31 to 0.53, *p* < 0.01), and PRO (*r* ranged from 0.29 to 0.52, *p* < 0.01). Correlations between the subscales and SSE across two waves were weak except the Self subscale at Wave 1 and the Society subscale in Wave 1 (*r* ranged from −0.11 to −0.07, *p* < 0.05). The subscales were also significantly correlated with the Respect Scale (*r* ranged from 0.33 to 0.67, *p* < 0.01), Care Scale (*r* ranged from 0.32 to 0.70, *p* < 0.01), Acceptance Scale (*r* ranged from 0.28 to 0.63, *p* < 0.01), and Integrity Scale (r ranged from 0.39 to 0.74, *p* < 0.01). Hypothesis 3b was accepted. However, the AVE values of all subscales were below 0.50, indicating that the indicators explained less than 50% of the variance in their respective factors, which does not meet the usual convergent validity criterion for AVE. Therefore, Hypothesis 3c was rejected. In short, there was solid support for the convergent validity of the total CRS, whereas evidence for the convergent validity of the subscales was mixed.

#### 3.3.4 Discriminant validity

The correlations between the subscales and the total scores, and the theoretically irrelevant items were mostly insignificant, and the few significant effects were small in magnitude |*r*| ≤ 0.20 (Table 8). Thus, Hypotheses 4a and 4b were accepted. The square roots of the AVE values were lower than the corresponding inter-factor correlations (Table 9), suggesting that each construct shared more variance with other factors than their own indicators; hence, suggesting the rejection of Hypothesis 4c. The HTMT ratios evidenced borderline discriminant validity: Self-Family (Wave 1 = 0.90; Wave 2 = 0.85), Self-Society (Wave 1 = 0.90; Wave 2 = 0.89), and Family-Society (Wave 1 = 0.90; Wave 2 = 0.90). Values approaching the conservative 0.90 threshold indicate distinct yet highly related factors, with improvement in Self-Family at Wave 2 supporting discriminant validity. Therefore, Hypothesis 4d was accepted.

**Table 8:** Pearson correlations between the CRS scores and four discriminant indicators (*N* = 963).

Discriminant items	Self	Family	Society	Self	Family	Society	Total	Total
	W1	W1	W1	W2	W2	W2	W1	W2
Do you like Korean movie (W1)?	0.04	0.10^**^	0.09^**^	0.01	0.08^**^	0.05	0.09^**^	0.06
Do you like playing basketball (W1)?	−0.04	0.05	0.04	−0.07^*^	0.01	0	0.03	−0.01
Do you agree that Hong Kong has a good climate (W1)?	0.06	0.09^**^	0.09^**^	0.02	0.03	0.05	0.09^**^	0.04
Do you like ice cream (W1)?	0.13^**^	0.10^**^	0.06	0.07^*^	0.04	0	0.10^**^	0.04
Do you like Korean movie (W2)?	0.02	0.09^**^	0.06	0.05	0.11^**^	0.06^*^	0.07^*^	0.09^**^
Do you like playing basketball (W2)?	−0.01	0.04	0.06	−0.03	0.06	0.07^*^	0.04	0.05
Do you agree that Hong Kong has a good climate (W2)?	−0.01	0	0.01	0	−0.01	0.01	0	0
Do you like ice cream (W2)?	0.10^**^	0.08^*^	0.11^**^	0.20^**^	0.15^**^	0.14^**^	0.10^**^	0.17^**^

^**^ Correlation is significant at the 0.01 level (2-tailed). ^*^Correlation is significant at the 0.05 level (2-tailed).

**Table 9:** Standardized factor loadings and latent factor correlations from CFA (*N* = 963).

Latent variables and HTMT	Fit indices							
χ^2^	1,425.97							
df	356							
*p*	< 0.001							
TLI	0.90							
CFI	0.91							
RMSEA	0.06							
SRMR	0.05							
**Standardized factor loadings**								
**Factors**	**Item number and content**				**Wave 1**		**Wave 2**	
Self	Item 1				0.60		0.62	
	Item 8				0.59		0.63	
	Item 13				0.65		0.67	
	Item 16				0.73		0.76	
	Item 19				0.69		0.72	
Family	Item 2				0.56		0.57	
	Item 4				0.59		0.59	
	Item 6				0.74		0.76	
	Item 7				0.57		0.59	
	Item 10				0.73		0.74	
	Item 12				0.51		0.52	
	Item 14				0.73		0.74	
	Item 17				0.73		0.74	
	Item 20				0.82		0.81	
Society	Item 3				0.51		0.52	
	Item 5				0.67		0.70	
	Item 9				0.70		0.73	
	Item 11				0.61		0.61	
	Item 15				0.62		0.62	
	Item 18				0.71		0.76	
**AVE for the total scale and each factor, and inter-factor correlations**								
	**Wave 1**				**Wave 2**			
Factors	1	2	3	AVE	1	2	3	AVE
1. Self	1			0.43	1			0.47
2. Family	0.88	1		0.45	0.83	1		0.46
3. Society	0.88	0.86	1	0.41	0.88	0.86	1	0.44
HTMT	Wave 1				Wave 2			
Self–Family	0.90				0.85			
Self–Society	0.90				0.89			
Family–Society	0.90				0.90			

AVE, average variance extracted. All the loadings and correlation coefficients were significant at the level of *p* < 0.001.

In short, there was mixed but overall adequate support for the discriminant validity of the total scale and subscales.

#### 3.3.5 Reliability

Reliability estimates (Table 10) demonstrated excellent internal consistency across both waves. Composite reliability values exceeded 0.94 for all subscales, while Cronbach's α of the subscales (0.78 − 0.88) and McDonald's ω(0.78 − 0.88) met acceptable thresholds (>0.70), with mean inter-item correlations (0.40 − 0.47) indicating adequate item convergence. The total scale's reliability also met the thresholds. Therefore, Hypotheses 5a–5c were accepted.

**Table 10:** Reliability of the CRS across two time points (*N* = 963).

Indices	Self	Family	Society	Total scale
Wave 1				
Composite reliability	0.94	0.96	0.95	
Cronbach's α	0.78	0.88	0.80	0.93
McDonald's ω	0.78	0.88	0.80	0.93
15.6-7.5,-14.1242ptMean inter-item correlation	0.42	0.45	0.40	0.40
Wave 2				
Composite reliability	0.95	0.96	0.95	
Cronbach's α	0.81	0.88	0.82	0.93
McDonald's ω	0.81	0.88	0.83	0.93
Mean inter-item correlation	0.47	0.46	0.43	0.42

In sum, there was evidence for the factorial, convergent, and discriminant validity, as well as measurement invariance and reliability, of the CRS.

## 4 General discussion

With reference to the above-mentioned gaps, this study offers several distinctive contributions. First, it integrates both Western and Chinese conceptions of responsibility in the development of the CRS by explicitly incorporating responsibility to family. Second, it rigorously evaluates the scale's content validity alongside comprehensive psychometric characteristics, including factorial validity, measurement invariance, convergent validity, discriminant validity, and reliability. Third, it analyzed the CRS's content validity and psychometric performance among Hong Kong university students in a Chinese context which is novel in nature. Fourth, the longitudinal design has addressed the lack of invariant responsibility scales in the existing scientific literature. Finally, the large longitudinal sample size is another unique feature of the study.

The findings generally supported the three-factor structure of the CRS, comprising dimensions of self, family, and society responsibility, with only a few item residuals correlated which can be regarded as reasonable. Notably, the correlated observed variables shared wording such as “knowing,” “important,” “willing,” and “harmony.” These residual correlations may indicate local dependence, suggesting that these items share additional variance beyond what is captured by the three latent factors. This local dependence may reflect wording-related variance. More specifically, the shared wording appears to reflect themes of self-awareness and personal willingness. In this sense, responsibility may involve not only fulfilling obligations across the domains of self, family, and society, but also recognizing these obligations, understanding their importance, and willingly endorsing them. For example, recognizing the importance of harmony in both family and society may enhance individuals' capacity to fulfill their responsibilities. This interpretation is broadly consistent with prior work by Lio et al. (2023), finding that among seminary students, self-awareness was positively correlated with the acceptance of personal responsibility. Likewise, individuals need clarity about their roles within the family and society in order to know how to act responsibly, and awareness of family expectations is central to becoming a responsible person. This returns to the key role of self-awareness in fulfilling responsibility and aligns, to some extent, with Western views that autonomous personal commitment lies at the core of responsibility (Hill Jr, 1994). Several scholars, such as Hieronymi (2014) and Stout (2019), have emphasized the importance of emotional awareness and reflective engagement with one's own agency in taking responsibility for actions and attitudes, highlighting that reflective self-evaluation is deeply entwined with responsible agency.

In summary, because the residual correlations involved only a small number of items and were specified on the basis of shared wording rather than introduced indiscriminately, they do not appear to undermine the overall three-factor structure. Nevertheless, these correlations should be interpreted with caution, as they provide suggestive rather than conclusive evidence of shared semantic themes. Items that share semantic themes–such as awareness of one's role– can be seen as capturing the holistic nature of responsibility in collectivist contexts, suggesting an underlying emphasis on self-awareness as a bridge between personal, familial, and societal obligations.

Although family and society typically have different personal meanings, the discriminant validity of the family factor vs. the society factor was only marginal. The factor correlations between the self, family, and society factors were also high. This aligns with traditional Chinese values, in which there is no sharp boundary between self, family and society. In Chinese culture, family and societal domains often carry overlapping personal meanings, as family obligations extend into societal roles through Confucian principles such as filial piety (孝; xiao) and collective harmony. In contrast, time and energy devoted to family or self-care in the Western contexts are often seen as competing with efforts to contribute to society as an employee, giving rise to the notion of work-life balance as a way to manage conflicts between work and non-work roles (Sirgy and Lee, 2018). In Chinese philosophy, one needs to be capable of regulating themselves, achieving family harmony and only then becoming capable of governing or unifying the country (修身齐家治国平天下; xiu shen, qi jia, zhi guo, ping tian xia) . In other words, social contribution is thus viewed as an outgrowth of the capability of running or ruling one's family. Cheng et al. (2021)'s research in Hong Kong shows that family cohesion is longitudinally associated with participants' perception of social trust, which in turn predicts perceived social responsibility, underscoring the pathway from family dynamics to civic orientation. Similarly, clan culture–grounded in extended kinship networks and obligations–was also found to contribute to the social responsibility of the business owner in the Chinese context (Xu and Guo, 2024). Taken together, the present psychometric evidence and the prior literature suggest that the CRS is culturally grounded and well-suited to capture the developmental change in responsibility among Chinese participants.

One of the interesting findings is that egocentrism was insignificantly or only weakly associated with the CRS subscales and the total score. This pattern suggests that, at least in this sample, being more egocentric, such as believing that one's own feelings are more important than others', does not necessarily undermine self-reported, role-based senses of duty to family, community, and society, which may be framed as relational obligations rather than as opposites of self-concern. It is consistent with Markus and Kitayama (2014)'s view that in collectivist cultural contexts, people often experience the self as intertwined with close others and groups, so that personal feelings and close group interests are partially merged rather than in zero-sum conflict. It also resonates with Duclos and Barasch (2014)'s finding that, for participants from collectivist cultures, helping in-group members strengthens the belief that such helping contributes to their own happiness, which in turn promotes subsequent benevolent behavior.

The weak association between egocentrism and responsibility can be further understood through the dual-process model theory (Moore and Loewenstein, 2004), which distinguishes between a fast, automatic system and a slower, controlled system of information processing. It is plausible that responsibility judgments and behaviors rely more heavily on the controlled, reflective system, whereas egocentric tendencies are more closely tied to automatic, instinctive processes. Emerging adults may still be substantially dependent on automatic processes in everyday decision making, while gradually strengthening more reflective, rational behavioral choices over time (Gibbons et al., 2009). According to the critical review by Tang (2019) on attitudes toward work in Chinese Generation Z, there has been a significant trend toward individualism, while Confucian cultural roots remain strong in this population. Therefore, a willingness to endorse and enact responsibilities in their key roles among Generation Z emerging adults may not be strongly associated with low levels of egocentrism.

The findings of this study supported the hypothesis that the CRS is consistent over time, providing evidence for its temporal stability. Although prior research has developed responsibility scales for Chinese university students (Ren et al., 2023; Liu et al., 2020; Wong and Liang, 2020), the measurement invariant property of responsibility has not been well-explored. The strong measurement invariance of the CRS reported in the current study adds to the present literature and supports the hypothesis that the CRS evaluates the same responsibility concept at both time points. Accordingly, the CRS can be used to evaluate developmental changes in subjective personal responsibility from late adolescence through emerging adulthood and into the working years, particularly during early career transitions, when individuals often experience substantial shifts in worldview and life orientation (Vinken, 2007). The scale demonstrated metric measurement invariance across gender and local/non-local status, indicating that the factor structure and factor loadings are equivalent across these groups. However, full scalar invariance was not supported, as constraining item intercepts significantly worsened model fit, suggesting that item thresholds may differ between males and females and between local and non-local subgroups. Consequently, the scale supports invariant factor loadings across gender and local/non-local status, but latent mean comparisons should be interpreted with caution or based on partial scalar invariance rather than assuming full equivalence. This implies that group differences may reflect both true variations in the construct and differences in how items are perceived.

## 5 Strengths and limitations

First, this is the first known study to examine responsibility across self, family, and societal domains. Previous scales (Liu et al., 2020; Ren et al., 2023) and theoretical articles (Jønsson and Fasano, 2025) have mainly focused on one aspect of responsibility or framed it from a Western perspective. Second, the present research conducted content validation and a longitudinal survey to rigorously test the CRS's psychometric properties, establishing a solid theoretical and empirical foundation. Previous scales only tested the psychometric properties in a cross-sectional way based on limited samples (Liu et al., 2020; Ren et al., 2023). Longitudinal reliability and validity analyses provide evidence that the CRS can measure developmental changes in responsibility. Third, the research comprehensively evaluated factorial, convergent, and discriminant validity, measurement invariance, and reliability, demonstrating the CRS's psychometric rigor. The scale is important for the development of positive youth development programs to enhance the wellbeing and leadership attributes of university students (Shek, 2010) and adolescents (Tan et al., 2025; Zhu et al., 2025). Furthermore, this scale contributes to an alternative perspective when evaluating university student's psychosocial outcomes. For program evaluation, such as service leadership education programs which have shown effectiveness in promoting life satisfaction (Shek et al., 2023), it is worthwhile to apply CRS to gauge its effectiveness and to explore the influence of service leadership on such virtue.

Although the study is ground-breaking, it has several limitations. First, it was administered only to Hong Kong university students via convenience sampling, which might potentially be the reason that the item score's ranges are high. However, as our sample was based on ten universities in Hong Kong, its representativeness is high. We followed rigorous scale development process and showed that the CRS has strong psychometric performance. Given sociocultural differences between Hong Kong and Mainland China, future research should test CRS invariance across rural and urban Mainland Chinese areas to identify potential biases. Second, the study relied solely on self-reports. Future studies should incorporate other informants' perspectives to strengthen evidence of convergent validity. Third, we did not examine criterion validity due to the limitation in research design. Future research could examine the CRS better predicts established benchmark variables comparing with similar scales within and outside educational settings.

## 6 Conclusions

This pioneering study assessed the content validity, factorial validity, measurement invariance, convergent and discriminant validity, and longitudinal reliability of the CRS in university students in Hong Kong, China. Grounded in a solid conceptual framework and rigorous development process, the findings affirm the factorial validity, convergent and discriminant validity, measurement invariance, and reliability of the CRS. Thus, the CRS serves as a reliable instrument for measuring the self-reported sense of responsibility among university students in contemporary Chinese settings. With its robust psychometric qualities, this research suggests that the CRS can support training and intervention initiatives by tracking the development and progress of responsibility over time.

## Data Availability

The raw data supporting the conclusions of this article will be made available by the authors, without undue reservation.
