# Supplementary material for: Psychometric properties of the Chinese Responsibility Scale in university students in Hong Kong, China
Source: Front Psychol. 2026 Jun 25;17:1843854. doi: 10.3389/fpsyg.2026.1843854 (PMC13345856; doi:10.3389/fpsyg.2026.1843854)
Supplement: Supplementary file 1 [file Data_Sheet_1.pdf]

Table S1

Item Content in Traditional Chinese and English

1. 我明白提升自我修養的重要性。

I understand the importance of improving my self-cultivation.

2. 我了解家人對自己的期望。

I understand my family's expectations of me.

3. 我了解自己在社會中所扮演的角色。

I understand the role I play in society.

4. 我了解自己在家所扮演的角色。

I understand the role I play in my family.

5. 我贊同每個人都應該關心社會。

I agree that everyone should care about society.

6. 我覺得承擔家庭責任是很重要的。

I think it is very important to take on family responsibilities.

7. 家庭和諧是重要的。

Family harmony is important.

8. 每個人都應該遵守社會規範。

Everyone should follow social norms.

9. 我關心我所做的事會否對社會帶來正面影響。

I care about whether what I do has a positive impact on society.

10. 我樂意分擔家庭成員的煩惱。

I am willing to share my family members’ worries.

11. 我樂意看到一個和諧的社會。

I am happy to see a harmonious society.

12. 我經常幫助家人做家務。

I often help my family with household chores.

13. 每個人應該對自己的言行負責。

Everyone should take responsibility for their words and actions.

14. 我樂意照顧家人的情緒。

I am willing to take care of my family members’ emotions.

15. 我經常關心社會發生的事。

I often care about what happens in society.

16. 我樂意改善自己以更好地履行自己的職責。

I am willing to better myself to fulfill my responsibilities more effectively.

17. 我樂意照顧年長的父母。

I am willing to take care of my elderly parents.

18. 我樂意為我的社區做一些事以讓它變得更好。

I am willing to do things for my community to make it better.

19. 我會盡力做好自己的份內事。

I will do my best to carry out my duties.

20. 我樂意承擔家庭的責任。

I am willing to take on family responsibilities.
